# Effect of Mass Supplementation with Ready-to-Use Supplementary Food during an Anticipated Nutritional Emergency

**DOI:** 10.1371/journal.pone.0044549

**Published:** 2012-09-12

**Authors:** Emmanuel Grellety, Susan Shepherd, Thomas Roederer, Mahamane L. Manzo, Stéphane Doyon, Eric-Alain Ategbo, Rebecca F. Grais

**Affiliations:** 1 Epicentre, Paris, France; 2 Médecins Sans Frontières, Paris, France; 3 Regional Department of the Ministry of Health, Maradi, Niger; 4 United Nations International Children Emergency Fund, Niamey, Niger; Aga Khan University, Pakistan

## Abstract

**Background:**

Previous studies have shown the benefits of ready-to-use supplementary food (RUSF) distribution in reducing the incidence and prevalence of severe acute malnutrition.

**Methods and Findings:**

To compare the incidence of wasting, stunting and mortality between children aged 6 to 23 mo participating and not participating in distributions of RUSF, we implemented two exhaustive prospective cohorts including all children 60 cm to 80 cm, resident in villages of two districts of Maradi region in Niger (n = 2238). Villages (20) were selected to be representative of the population. All registered children were eligible for the monthly distributions between July and October 2010. Age, sex, height, weight, and Mid-Upper Arm Circumference (MUAC) were measured at baseline and two weeks after each distribution; the amount and type of distribution and the amount shared and remaining were also assessed. We compared the incidence of wasting, stunting, and mortality among children participating in the distribution (intervention) of RUSF versus children not participating in the distribution (comparison). The absolute rate of wasting was 4.71 events per child-year (503 events/106.59 child-year) in the intervention group and 4.98 events per child-year (322 events/64.54 child-year) in the comparison group. The intervention group had a small but higher weight-for-length Z-score gain (−0.2z vs. −0.3z) and less loss of MUAC than the comparison group (−2.8 vs. −4.0 mm). There was no difference in length gain (2.7 vs. 2.8 cm). Mortality was lower for children whose households received the intervention than those who did not (adjusted HR 0.55, 95% CI: 0.32–0.98).

**Conclusions:**

Short-term distribution with RUSF for children 6 to 23 months improve the nutritional status of children at risk for malnutrition. Fewer children who participated in the RUSF distribution died than those who did not.

## Introduction

Young children are vulnerable to the growth, morbidity and mortality consequences of malnutrition. As a result, interventions targeting young children, specifically those 6 to 23 months [1], are an essential component for the prevention of malnutrition and associated death. Addressing this need led to the development of easily consumed complementary foods tailored to the specific needs of young children [2], [3].

Each year in Niger, the months preceding the harvest (June to October) are associated with increased wasting among children. The region of Maradi, located in the south-central part of the country bordering Nigeria, has some of the highest rates of malnutrition in Niger [4]. To mitigate the effects of the hunger gap, large-scale mass distributions have been conducted at various scales each year in Niger. Previous evaluations of these interventions have been shown to reduce the burden of severe acute malnutrition in this population [5].

From July to October 2010, in collaboration with the Ministry of Health, Médecins Sans Frontières (MSF) and Forsani distributed of RUSF to registered children aged 6 to 23 months living in the districts of Guidam Roumdji and Madarounfa in the region of Maradi. The World Food Program (WFP) also distributed protection rations to families during this period. Here, we report the findings of a prospective cohort designed to monitor these interventions. We present a comparison of the incidence of wasting, anthropometric changes and mortality in children aged 6 to 23 months who participated or did not participate in the monthly distributions over a four month follow-up period.

## Methods

### Ethics

The study protocol was approved by the Comité Consultatif National d’Ethique du Niger, and authorized by the Ministry of Public Health of Niger. Approval from all heads of selected villages was received prior to the start of the study, and the objectives of the study and study protocol were explained to heads of households with eligible children before inclusion. An informed consent statement was read aloud in the local dialect before being signed or fingerprinted by the head of household or child care giver. Participation in distributions was not a pre-condition for obtaining free medical services; it was clearly stated that participants were free to withdraw from the study at any time.

### Setting

After a community awareness campaign, a mass registration exercise was held in July 2010 to enroll children in the distribution program. All children 60 cm to 80 cm in length (approximately 6 to 23 months) resident in the districts of Guidam Roumdji and Madarounfa were invited to attend and be registered for the program. Using length/height as a proxy for a child’s age is the standard and usual practice in such contexts [5]. Once registered, children were eligible to receive monthly distributions from July to October of Plumpy’Doz® (Nutriset, Malaunay, France). The composition of Plumpy’Doz® is available in the following publication [6]. Registration for the distribution program was closed at the end of the mass registration process. Children of families absent during this process or choosing not to register for the distributions were not subsequently eligible for inclusion in the distribution.

The chosen formulation of RUSF was specifically developed for children of this age according to the manufacturer. Monthly distributions of RUSF were made in 4×325 g pots (4 pots = 1 monthly ration per child) at sites located within walking distance from each village. At the time of RUSF distribution, nutrition assistants also screened children in attendance for MUAC<115 mm or oedema and referred children to the closest nutritional treatment program when indicated. The RUSF distribution was sometimes accompanied by WFP’s family protection ration (Table 1).

**Table 1:** Description of distributions, July –October, 2010, Guidam Roumdji and Madarounfa Districts, Niger.

Total number in the cohort by type of rations and byround of distribution	1^st^ roundN = 2238	2^nd^ roundN = 2238	3^rd^ roundN = 2238	4^th^ roundN = 2238	TotalN = 8952
Number of children receiving RUSF, (%)					
Plumpy’Doz© (4 pots of 345 g)	1130 (50)	1147 (51)	1122 (50)	1105 (49)	4504 (50)
Number of family receiving protection ration, (%)					
8.3 kg CSB, oil, sugar^1^	1132 (51)	0 (0)	0 (0)	0 (0)	1132 (13)
50 kg cereals, 5 kg pulses, 2 kg oil^2^	83 (4)	1082 (48)	1131 (50)	166 (7)	2462 (28)

1 The RUSF distribution was coupled with the WFP’s family protection ration of 8kg of CSB, oil and sugar in July in Guidam Roumdji only.

2 In both districts in August and September, the family protection rations given were 50 kg cereal, 5 kg pulses and 2 kg of oil.

### Study Design

To follow the nutritional status of children who were registered in the distribution program (intervention group) and those who failed to be registered (comparison group), we randomly selected twenty villages (ten in each district). A complete list of villages and hamlets [7] in both districts was stratified; first by accessibility (presence of a health center, market, paved road and modern water point within a radius of 10 km), and second by administrative status (village or hamlet). Villages were randomly selected with probability proportional to population size within the four strata.

Two days after registration was closed, exhaustive enrolment was conducted independently by the study teams in each of the 20 cohort villages of all children meeting the inclusion criteria of the MSF/Forsani distribution program by teams going from house-to-house within each village. Thus, within the cohorts, there were both children who were registered to receive the distribution and those that were not.

### Measurements

Approximately two weeks after each monthly distribution, trained nutrition assistants, independent of the staff conducting the distributions, carried out anthropometric measurements with the use of standardized methods and calibrated instruments. Study teams resided in the cohort villages for a minimum of 2 days per month during data collection. Child height (recumbent length if <87 cm) was measured to the nearest 0.1 cm using a Shorr® wooden measurement board. Weight was measured to the nearest 0.1 kg using a hanging Salter scale. MUAC was measured at the midpoint of a child’s left arm with a plastic measuring tape with a precision of 1 mm. Bilateral oedema was diagnosed if an imprint was observed after 5-second pressure with the thumb on the dorsum of both feet. Any child found with weight-for-length (height) Z score (WLZ) <-3 of the World Health Organisation growth standards or with a MUAC <115 mm or bilateral oedema or medical complications at a follow-up visit was referred to the nutritional program or neighboring governmental health facility for treatment provided at no cost.

During the first and last post-distribution visits, a standardized questionnaire was administered to obtain information on household, maternal and child socio-demographic characteristics. We estimated child age at enrolment using a special event calendar if exact date of birth was unknown. An abridged questionnaire was used at each post-distribution visit to obtain information on the major health events including both death of the child and the extent of sharing of the RUSF and WFP ration within the household. Moreover, during each visit, the remaining quantities of distributed food were weighed and compared with the rations received.

### Statistical Analysis

Children aged 6 to 23 months at baseline in the cohort villages who were registered and participated in at least one of the four monthly distributions (intervention group) and those who did not participate in any of the RUSF distributions were compared (comparison group). Our endpoints were wasting (WLZ <-2 or MUAC <125 mm), severe wasting (WLZ <-3, or MUAC <115 mm), stunting (length-for-age (LAZ) <-2) severe stunting (LAZ <-3) and mortality. Mortality events included all reports for which the cause for absence from surveillance visits was reported to be death by a family member or the head of village.

We examined the distribution of baseline (July 2010) characteristics by status (intervention or comparison group) using generalized estimating equations to adjust standard errors for clustering at the village-level. Next, we explored the association between status and the incidence of wasting, stunting and mortality adjusting for potential differences between groups. Among children free from the outcome at baseline, we estimated hazard ratios (HR) and 95% confidence intervals (CI) using marginal Cox proportional hazards models with time from registration to the event (wasting, stunting, or death) as the outcome and months as the time scale. All 95% CIs used robust estimates of the variance to account for clustering at the village-level as well as a shared-frailty model as developed by Andersen Robert [8]. Children contributed person-time to the analysis from baseline (July 2010) until the first occurrence of the outcome or the end of follow-up (October 2010).

To control for confounding in light of the relatively large number of potential confounders and limited events, we used propensity score adjustment [9], [10]. Additional details of this method have been published previously [11], [12]. Baseline characteristics considered to be related to the probability of registering or not registering for the distributions, and those variables that were different between the intervention and comparison groups at baseline by univariate analyses using *P*<0.2 were used as covariates. Covariates included child’s age group at baseline (<6, 6–11, 12–23, >23 mo), sex, length, baseline WLZ, LAZ and MUAC, administrative district, accessibility to health center (presence of a market, a modern water point supply and a main road). Scores were then computed in the full cohort with a logistic regression modeling this probability. Indicators for quartile categories of the propensity score were included as independent variables in each outcome model. Each propensity score was divided into quartile categories. When considering the potentially confounding effects of the covariates investigated here, there was no difference when using traditional multivariate or propensity score adjustment. Potential interactions were assessed with Cox models using partial likelihood ratio test for the wasting, stunting and mortality outcomes.

All data were collected on standardized forms and double entered into EpiData version 2.1 (EpiData Association, Odense, Denmark). Analyses were conducted using STATA version 10 (StataCorp, College Station, TX, USA).

## Results

A total of 2238 children aged 6 to 23 months in 2127 household were enrolled in the cohort at baseline. None of the children in the cohort communities refused to join the study. Of the children enrolled in the cohort 1400 children were registered to participate in the distribution (intervention) and 838 children (comparison) were not registered to receive the supplementary food distribution. The cohort represented 4.3% of the total 6 to 23 month old population of the districts of Guidam Roumdji and Madarounfa [7].

All children did not receive the same number or type of distributions over the 4 month follow-up. Although, approximately 51% of households received Corn Soy Blend (CSB) at the first distribution (Table 1) there were no further distributions of this type. Most families received only one or two distributions of the family rations (Table 2). Thirteen percent of the intervention group only received RUSF once, 9% twice, 19% three times and 59% received all 4 distributions; thus, 78% of the children designated as receiving the interventions (intervention group) received 3 or more distributions.

**Table 2:** The intervention group per number of distribution and type of rations received, July-October 2010, Guidam Roumdji and Madarounfa Districts, Niger.

Number of distributions received	Number of children receiving RUSF,N (%)	Number of families receivingprotection ration, N (%)	
	Plumpy’Doz© (4 pots of 345 g)	8.3 kg CSB, oil, sugar	50 kg cereals, 5 kg pulses, 2 kg oil
x1	187 (13)	1132 (100)	296 (23)
x2	129 (9)	0 (0)	855 (67)
x3	277 (20)	0 (0)	72 (6)
x4	807 (58)	0 (0)	60 (5)
Total distributions	4504 (81)	1132 (20)	2462 (44)
Total children/family	1400 (100)	1132(81)	1283 (91)

At baseline, the intervention and comparison group differed in age and household composition (Table 3). Children in the intervention group were slightly younger (p = 0.004) and lived in households containing more under 5 yr old children (p = 0.001). There were 5 (0.3%) children absent at the end of the study and whose outcome was therefore unknown in the intervention group and 35 (4.2%) in the comparison group. The number of children with anthropometry measured in July, August, September and October were 1392, 1364, 1328 and 1221 in the intervention group and 794, 760, 707, and 597 in the comparison group respectively. Over all distributions, 58% of RUSF was reported to be shared with other younger siblings (>110cm in height) within the same household (Table 4). Almost all of the family protection ration (85%) was shared within the same household.

**Table 3:** Characteristics of the intervention and comparison groups at baseline (July 2010), Guidam Roumdji and Madarounfa Districts, Niger.

	Intervention group	Comparison group	p-value
No. of children	1400	838	
Person time observed, mo	4,624	2,740	
Person time if no missing data, mo	4,755	3,026	
Child Characteristics, N^1^ (%)			
Child age, mo			0.004
<11	467 (33.4)	250 (29.8)	
12–23	765 (54.6)	446 (53.2)	
≥24	168 (12.0)	142 (17.0)	
Child Length			0.11
>60–≤70 cm	602 (43.5)	339 (40.5)	
>70–≤80 cm	791 (56.5)	499 (59.5)	
Gender			0.43
Male	690 (47.5)	398 (49.3)	
Female	710 (52.5)	440 (50.7)	
Wasting (WHO 2006)			
WLZ, mean (± SD)	−1.13 (1.0)	−1.08 (1.0)	0.24
Wasting (WLZ less than -2Z)	265/1400 (18.9)	146/838 (17.4)	0.20
Severe wasting (WLZ less than -3Z)	32/1400 (2.3)	27/838 (3.3)	0.11
Stunting (WHO 2006)			
LAZ, mean (± SD)	−2.54 (1.2)	−2.61 (1.3)	0.17
Stunting (LAZ less than -2Z)	926/1400 (66.1)	583/838 (69.6)	0.10
Severe Stunting (LAZ less than -3Z)	493/1400 (35.2)	303/838 (36.2)	0.34
MUAC			0.15
Less than 125 mm	234/1400 (16.7)	121/838 (14.4)	
Less than 115 mm	49/1400 (3.5)	22/838 (2.7)	
Household Characteristics, N (%)			
No of children younger than 5y at home			0.001
1	305 (22.3)	231 (30.5)	
2	756 (55.2)	435 (57.4)	
3	291 (21.2)	89 (11.8)	
≥4	17 (1.3)	3 (0.4)	

1 Sums may not add up to totals due to missing values.

**Table 4:** Reported sharing of RUSF and the protection ration within the family, July-October 2010, Guidam Roumdji and Madarounfa Districts, Niger.

Number of distributions received	1	2	3	4	Total
RUSF, N (%)					
No sharing reported^1^	90 (8)	262 (23)	235 (21)	202 (19)	789 (18)
Sharing with siblings <110cm of height^2^	341 (30)	337 (30)	514 (46)	607 (55)	1,799 (40)
Sharing within the family^3^	435 (39)	446 (39)	295 (27)	220 (20)	1,396 (31)
Sharing inside and outside the family	254 (23)	94 (8)	65 (6)	63 (6)	476 (11)
Protection ration, N (%)					
No sharing reported^1^	0 (0)	3 (0.3)	0 (0)	1 (0.6)	4 (0.2)
Sharing with siblings <110 cm of height^2^	0 (0)	0 (0)	0 (0)	0 (0)	0 (0)
Sharing with all the family^3^	83 (100)	941 (88.3)	962 (85.2)	97 (58.8)	2,083 (85.3)
Sharing inside and outside the family	0 (0)	121 (11.4)	167 (14.8)	67 (40.6)	355 (14.5)

1 No sharing was defined as the ration was reported to be consumed only by children 60 cm to 80 cm in length (target population).

2 Sharing with siblings <110 cm of height was defined as sharing siblings less than 110 cm of height (approximately 5 years of age).

3 Sharing within the family was defined as between children 60 cm to 80 cm in length, their siblings less than 110 cm of height and the rest of the family. A family or household was defined as the nuclear family.

The absolute rate of wasting was 4.71 events per child-year (503 events/1,280 child-months) in the intervention group and 4.98 events per child-year (322 events/775 child-months) in the comparison group. The intervention group had a small but higher WLZ change (−0.2 vs. −0.3 z; p = 0.006) and less loss of MUAC than the comparison group (−2.8 vs. −4.0mm; p = 0.002) comparing pre- to post-final distributions. There was no difference in length gain (2.7 vs. 2.8cm) among groups (Table 5). Fewer initially non-wasted children developed moderate wasting in the intervention group than the comparison group (Table 6).

**Table 5:** Change in anthropometry of the intervention group and comparison group between baseline and two weeks post final distribution, July-October 2010 Guidam Roumdji and Madarounfa Districts, Niger.

Child at recruitment vs. last visit	Intervention group	Comparison group	p-value
Anthropometric gains, N (95%, CI)			
MUAC, mm	−2.8 (−3.2−2.3)	−4.0 (−4.7−3.3)	0.002
WLZ, score	−0.2 (−0.2−0.2)	−0.3 (−0.4−0.3)	0.006
Weight, g	395 (364–425)	327 (281–372)	0.05
Length, cm	2.7 (2.6–2.8)	2.8 (2.6–2.9)	0.24

**Table 6:** Effect of supplementation on the incidence of wasting, stunting^1^ and morality between the first distribution and 2 weeks after the last distribution (97 to 101 days), July-October 2010 Guidam Roumdji and Madarounfa Districts, Niger.

Measure	Intervention group	Comparison group	p-value
Wasting (WHO 2006)			0.05
N^2^	1135	692	
Number of events/child-month at risk	503/1280	322/775	
Incidence rate per 100 child-month (95% CI)	3.92 (3.62–4.31)	4.15 (3.74–4.63)	
Unadjusted HR (95% CI)	0.96 (0.81–1.14)	1.00 (reference)	
Adjusted HR^3^ (95% CI)	0.84 (0.71–0.99)	1.00 (reference)	
Severe wasting (WHO 2006)			0.37
N^2^	1368	811	
Number of events/child-month at risk	97/1598	61/941	
Incidence rate per 100 child-month (95% CI)	6.09 (4.91–7.33)	6.48 (5.13–8.33)	
Unadjusted HR (95% CI)	0.96 (0.63–1.43)	1.00 (reference)	
Adjusted HR^3^ (95% CI)	0.83 (0.56–1.24)	1.00 (reference)	
MUAC less than 125mm			0.56
N^2^	1166	717	
Number of events/child-month at risk	624/1320	411/806	
Incidence rate per 100 child-month (95% CI)	47.3 (43.6–51.1)	50.1 (46.2–56.1)	
Unadjusted HR (95% CI)	1.15 (1.02–1.31)	1.00 (reference)	
Adjusted HR^3^ (95% CI)	1.04 (0.91–1.17)	1.00 (reference)	
MUAC less than 115mm			0.44
N^2^	1351	816	
Number of events/child-month at risk	105/1602	68/945	
Incidence rate per 100 child-month (95% CI)	6.55 (5.44–8.00)	7.19 (5.62–9.01)	
Unadjusted HR (95% CI)	0.95 (0.64–1.43)	1.00 (reference)	
Adjusted HR^3^ (95% CI)	0.85 (0.57–1.28)	1.00 (reference)	
Stunting			0.87
N^2^	474	255	
Number of events per child-month	144/536	42/291	
Incidence rate per 100 child-month (95% CI)	26.8 (22.8–31.6)	14.4 (10.7–19.5)	
Unadjusted HR (95% CI)	1.77 (1.13–2.77)	1.00 (reference)	
Adjusted HR^3^ (95% CI)	0.96 (0.61–1.52)	1.00 (reference)	
Severe stunting			0.98
N^2^	907	535	
Number of events per child-month	145/1067	45/623	
Incidence rate per 100 child-month (95% CI)	13.6 (11.5–15.9)	7.21 (5.37–9.73)	
Unadjusted HR (95% CI)	1.79 (1.14–2.81)	1.00 (reference)	
Adjusted HR^3^ (95% CI)	1.00 (0.62–1.62)	1.00 (reference)	
Mortality			0.03
N^2^	1400	838	
Number of events/child- month at risk	27/1678	24/994	
Incidence rate per 100 child-month (95% CI)	1.61 (1.10–2.37)	2.41 (1.55–3.62)	
Unadjusted HR (95% CI)	0.70 (0.40–1.21)	1.00 (reference)	
Adjusted HR^3^ (95% CI)	0.55 (0.32–0.98)	1.00 (reference)	

1 Wasting and severe wasting are defined as WLZ<−2 and WLZ <−3 and stunting and severe stunting are defined as LAZ <−2 and LAZ <−3, respectively. Two children who had oedema were not included in analyses of wasting or stunting but were included in the morality analysis.

2 Number of children contributing to unadjusted analysis.

3 From marginal Cox proportional hazards models, where the outcome variable is time until first event and time is calendar month. Predicators in the adjusted model included distribution type and indicators for quartiles of the estimated propensity score. The propensity score was estimated using logistic regression where the probability of receiving the RUSF supplementation strategy was predicted given child’s age at baseline (<6, 6–11, 12–23, ≥24 mo), sex, length, village district, accessibility to health center, market, modern water supply, main road, baseline MUAC, WLZ and LAZ (continuous), management of severe acute malnutrition, number of children under five years in the household and if RUSF or the protection ration families was shared within the household.

Mortality was lower for children whose households were in the intervention group than those who were not (adjusted HR: 0.55, 95% CI: 0.32 to 0.98) (Table 4). In total, 29 per 1000 children enrolled in the cohort died during the follow-up period. Of these, no child receiving all 4 distributions died; 5 children died who received 3 distributions; 7 children receiving 2 distributions and 6 children receiving only one distribution (Table 7). Table 8 shows the antecedent nutritional status of children who died per 1000, for the intervention group and comparison group and Table 9 shows the absolute number of deaths by number of distributions received.

**Table 7:** Mortality of the intervention group and the comparison group by number of distributions received, July-October 2010 Guidam Roumdji and Madarounfa District, Niger.

Intervention group vs. Comparison group	1 vs. 0	2 vs. 0	3 vs. 0	4 vs. 0
Death per 1000	6 vs. 29	7 vs. 29	5 vs. 29	0 vs. 29
Unadjusted HR (95% CI)	1.97 (0.93–4.18)	2.86 (1.38–5.92)	1.07 (0.48–2.40)	–
Adjusted HR (95% CI)	1.99 (0.95–4.18)	1.96 (0.78–4.94)	0.73 (0.27–1.99)	–
p-value	0.07	0.15	0.55	–

**Table 8:** Antecedent nutritional status of the child at the last visit before death, July-October 2010 Guidam Roumdji and Madarounfa Districts, Niger.

Intervention group vs. Comparison group	Death per 1000	Unadjusted HR (95% CI)	p-value
MUAC ≥135 mm	8 vs. 23	0.34 (0.17–1.08)	0.06
MUAC ≥125 and <135 mm	29 vs. 32	0.91 (0.39–2.17)	0.90
MUAC <125 mm	25 vs. 43	0.58 (0.35–1.65)	0.34
WLZ ≥−2 Z	13 vs. 23	0.56 (0.03–3.08)	0.56
WLZ ≥−3 and <−2 Z	52 vs. 59	0.88 (0.39–2.89)	1.00
WLZ <−3 Z	31 vs. 74	0.42 (0.22–1.12)	0.54

**Table 9:** Absolute number of deaths by number of distribution received according to the nutritional status of children at the last visit, July-October 2010 Guidam Roumdji and Madarounfa Districts, Niger.

Number of distributions received	0	1	2	3	p-value
MUAC status before death,N (%)					0.26
≥135 mm	9 (37.5)	3 (33.4)	0 (0.0)	2 (25.0)	
≥125 and <135 mm	9 (37.5)	4 (44.4)	7 (70.0)	4 (50.0)	
≥115 and <125 mm	6 (25.0)	1 (11.1)	2 (20.0)	2 (25.0)	
<115 mm	0 (0.0)	1 (11.1)	1 (10.0)	0 (0.0)	
WLZ status before death,N (%)					0.66
≥−2 Z	15 (62.5)	6 (66.7)	4 (40.0)	4 (50.0)	
≥−3 and <−2 Z	7 (29.2)	3 (33.3)	6 (60.0)	3 (37.5)	
<−3 Z	2 (8.3)	0 (0.0)	0 (0.0)	1 (12.5)	

## Discussion

The results of this study show that distributions including an RUSF for children 6 to 23 months and a family protective ration had a modest but positive effect on prevention of wasting and anthropometric status. Importantly, deaths were halved for those who received the supplements compared to those who did not.

To our knowledge, this is the first study to document the benefits of distribution programs with RUSF in terms of mortality in this context. A previous randomized controlled trial conducted in the same two districts showed a marked effect on wasting and a moderate effect on stunting. However, a larger amount (500 kcal/d vs. 250 kcal/d) of a similar, but different, product (ready to use therapeutic food, Plumpy’nut®) was used. Although results suggested lower mortality, too few deaths were recorded to reach significance [13].

RUSFs are formulated to supply all of the essential nutrients; both those required to maintain body function and for normal growth [4], [14]. A deficiency of one or several of the functional nutrients impairs physiological or immunological function without any effect on anthropometric indices. Benefits in terms of mortality, combined with a very modest effect upon weight and MUAC may potentially have been due to the correction of functional deficiencies, not causally associated with anthropometric deficits, but resulting in functional changes increasing mortality risk [4]. It is noteworthy that many of the deaths were in children that were neither moderately or severely malnourished anthropometrically, and it appeared that this group of not-wasted children benefited most from the RUSF distribution in terms of mortality avoidance. This was unexpected and would indicate that even modest amounts of those nutrients whose deficiency is not associated particularly with wasting could be implicated in the reduction in mortality. This would have major implications for targeting in such situations, and perhaps for the composition of the RUSF supplied.

There are several important limitations to these results which require discussion. First, the selection of the villages to study was taken at random and fairly represents the population at large although children themselves were not randomized to receive the distributions, but were either registered or failed to be registered at the time of the initial mass-registration and subsequently observed. It is unclear why some children were not registered initially; possibly caretakers were absent at the time of registration, the benefits of the program were not adequately explained or advertised, or they felt their child did not need the RUSF on offer. As a result, differences between the intervention group and comparison group could account for the observed reduction in mortality. However, in addition to accounting for differences in the statistical analyses, baseline anthropometry of children was not significantly different between groups. The intervention group had a slightly lower, but not statistically different, mean weight-for-length, came from larger families and were younger. These are recognized risk factors for mortality; thus, the children receiving the distributions were likely to have been at higher risk of death than the comparison group. It is important to note that the population was under very severe stress with mortality rates when expressed in conventional emergency terms of 1.6/10,000/d for the intervention group and 2.7/10,000/d for the comparison group. As children identified as severely malnourished were admitted to therapeutic programs, in the absence of the distribution program mortality may have been higher. In addition, mortality in the comparison group may be underestimated; five children in the intervention group and 35 in the comparison group were lost to follow-up. If all, or a proportion, of these children were lost to follow-up because of death, the strength of the reduction in mortality with the distribution would increase. Overall, there were fewer deaths among children in the intervention group irrespective of the number of distributions received.

Second, there may be unexplained differences between the intervention and comparison group. One possible hypothesis arising from these results is that families receiving the distributions have children already showing signs of deterioration, as evidenced by the presence of known risk factors at baseline. Families with children who are in better health at the time of registration may chose not to participate highlighting the potential weakness of programs with closed enrollment. Although all families with children with heights equivalent to children aged 6 to 23 months were eligible for the distributions and nutritional programs if admission criteria were met, further research and improvements in terms of program awareness, acceptability and accessibility are needed. Furthermore, it is clearly an error to apply closed registration strategies in regions with a high background mortality and undernutrition. Operationally feasible strategies allowing for open registration for distributions should be developed in order to maximize coverage.

Third, it is possible that the severity of the situation was the reason for the extensive sharing of supplement within the family and this in turn led to the modest differences in wasting found, despite observed differences in mortality which has not been adequately documented elsewhere to our knowledge [15]. Nevertheless, even small changes in MUAC or weight may be of clinical significance for those who are already in the lower tail of the distribution of nutritional status [16], [17]. Increased energy intake has previously been associated with increased weight gain [18], [19], and the energy provided by RUSF is within the range (200 to 300 kcal/day, assuming average breast milk intake and sharing within the family) that older infants require from complementary foods [20]. Previous evaluations of RUSF supplementation have been consistent in demonstrating improved weight gain in a variety of study populations and against a range of comparator products, including micronutrient fortified flours [21], [22] and porridge [23].

Fourth, over the four month follow-up, we did not observe an effect upon stunting. Review of complementary feeding interventions suggests that the effect of RUSF on linear growth has been inconsistent, with significant improvements achieved only in some settings [24] and the acceleration of length gain may only occur after supplementation has been given for several months [25].

Fifth, it was not possible to differentiate the effect of the RUSF from the family protection rations, nor was it the aim of this study. However, the distribution of protective rations was inconsistent and almost non-existent during the fourth distribution. This coupled with the known inadequacies of nutrient composition of the family ration to meet the needs of young children contribute to the limited evidence for including an RUSF in distributions. Finally, potential errors in the child’s age at recruitment or measurement errors for the anthropometric variables, despite continual training of field teams, may have reduced or increased the statistical power to detect significant effects.

It is important to highlight that the cornerstone of all medical interventions is the early and appropriate treatment of children at risk of death, irrespective of the cause. Although formal verbal autopsies were not conducted in this study, parents reported the cause of death of their child to be malaria or fever in almost all instances. Children in our cohort benefitted from a comprehensive pediatric care package and were referred for nutritional treatment if they met the inclusion criteria for nutritional programs operating in the two districts. Participation in distribution programs provided advantages beyond that of the rations received and may have led families to seek prompt medical treatment for other conditions. However, it is important to emphasize that all families, whether they received or did not receive the distributions were screened between each distribution and referred for free and comprehensive medical care and rescue facilities; the mortality rate in the villages that were not included in this cohort study could thus have been substantially higher.

In conclusion, the results of this study show that the RUSF distribution with a protection ration for the families had a positive effect on wasting and anthropometric status of children who received the distribution in comparison to those who did not. Importantly, deaths were halved for recipients compared to non-recipients. These results suggest that with similar access to health services, distributions can have a positive impact on child survival. Contextual factors will continue to be important in determining the dose, duration, period and modalities of such preventive intervention based on RUSF. Dietary supplementation with foods specifically formulated for vulnerable populations have become a component of government-run social safety net programs [26]. In settings of endemic malnutrition and high child mortality, the health impacts of RUSF documented through humanitarian projects may help inform decision making for longer term programming.
