# Correction: Effect of Mass Supplementation with Ready-to-Use Supplementary Food during an Anticipated Nutritional Emergency

**DOI:** 10.1371/annotation/d41cce68-f8a3-45f1-beed-c2daaa938b88

**Published:** 2013-11-07

**Authors:** Emmanuel Grellety, Susan Shepherd, Thomas Roederer, Mahamane L. Manzo, Stéphane Doyon, Eric-Alain Ategbo, Rebecca F. Grais

In Table 6, a unit conversion problem has resulted in incorrect table values. Please find a correct version of the table here: 

**Figure:**
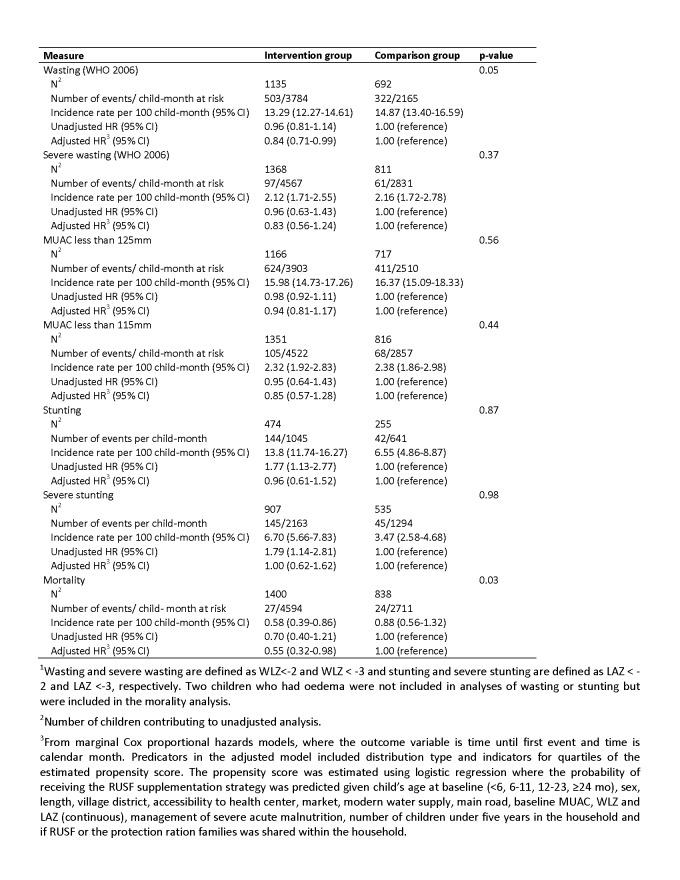


Necessary corrections to the text are as follows:

There is an error in the sixth sentence of the "Methods and Findings" portion of the Abstract. The correct sentence reads: The absolute rate of wasting was 1.59 events per child-year (503 events/315.3 child-year) in the intervention group and 1.78 events per child-year (322 events/180.4 child-year) in the comparison group.

There is an error in the first sentence of the fourth paragraph of the Results section. The correct sentence reads: The absolute rate of wasting was 1.59 events per child-year (503 events/3,784 child-months) in the intervention group and 1.78 events per child-year (322 events/2,165 child-months) in the comparison group.

The first sentence of the fifth paragraph of the results section should reference Table 6 in place of Table 4. The correct sentence reads: Mortality was lower for children whose households were in the intervention group than those who were not (adjusted HR: 0.55, 95% CI: 0.32 to 0.98) (Table 6).

There is an error in the eighth sentence of the fourth paragraph of the Discussion section. The correct sentence reads: It is important to note that the population was under very severe stress with mortality rates when expressed in conventional emergency terms of 1.7/10,000/d for the intervention group and 2.9/10,000/d for the comparison group. 

